# Musculoskeletal ultrasound in pediatric rheumatology

**DOI:** 10.1186/1546-0096-9-25

**Published:** 2011-09-12

**Authors:** Fatih Tok, Erkan Demirkaya, Levent Özçakar

**Affiliations:** 1 İskenderun Military Hospital, Physical Medicine and Rehabilitation Service, Hatay, Turkey; 2 Gülhane Military Medical Academy, Department of Pediatrics, Division of Pediatric Nephrology & Rheumatology, Ankara, Turkey; 3 Hacettepe University Medical School, Department of Physical Medicine and Rehabilitation Ankara, Turkey

**Keywords:** Musculoskeletal ultrasound, pediatrics, rheumatology

## Abstract

Although musculoskeletal ultrasound (MSUS) has emerged as an indispensible tool among physicians involved in musculoskeletal medicine in the last two decades, only recently has it become more attractive to pediatric rheumatologists. Thereafter, the use of MSUS in pediatric rheumatology has started to increase. Yet, an ever-growing body of literature shows parity and even superiority of MSUS when compared to physical examination and other imaging modalities.

MSUS is suitable for examination of children of all ages and it has certain advantages over other imaging modalities; as it is cheaper, mobile, instantly accessible bedside, easy to combine with clinical assessment (interactivity) and non-invasive. It does not require sedation, which facilitates repetitive examinations. Assessment of multiple locations is possible during the same session. Agitation is rarely a problem and small children can be seated in their parents' lap or they can even play while being examined.

## Background

Although musculoskeletal ultrasound (MSUS) has emerged as an indispensable tool among physicians involved in musculoskeletal medicine in the last two decades, only recently has it become more attractive to pediatric rheumatologists. Thereafter, the use of MSUS in pediatric rheumatology has started to increase. Yet, an ever-growing body of literature shows parity and even superiority of MSUS when compared to physical examination and other imaging modalities [1].

Several noninvasive techniques have been proposed to assess articular involvement of the pediatric rheumatoid diseases; however, magnetic resonance imaging (MRI) and MSUS have come to the forefront. MSUS has a tremendous advantage over MRI in that the examination can be performed quite rapidly. Rheumatoid patients, especially the younger ones, are easily bored and cannot tolerate lying motionless on hard table for the time required for an MRI examination. In addition, during sonographic examination, the patient may move other extremities relatively freely, and the procedure does not require sedation. Cost and availability factors also strongly favor MSUS. The real time imaging capability of US allows dynamic assessment of joint and tendon movements, which can often aid the detection of structural abnormalities [2]. On the contrary, the most important disadvantage of MSUS seems to be its user-dependency. Accordingly, prompt use of MSUS requires experience and thus education. The inability to visualize pathologies inside the bones or at sites where it is not possible to position the probe (e.g. surrounding the 2nd to 4th metacarpophalangeal (MCP) joints) would be other less noteworthy disadvantages.

MSUS is most commonly used in the assessment of soft tissue disease or detection of fluid collection. It can also be used to visualize musculoskeletal structures, such as muscle, fascia, tendon, para-tenon, ligament, synovium/capsule, hyaline and costal cartilage, fibrocartilage, nerve and bone surfaces [3]. However, US waves cannot penetrate into bone; therefore imaging of intra-bone disease is not possible. MSUS can also be used for guidance of aspiration, biopsy, and injection treatment [4].

Most musculoskeletal work is performed using gray scale, which means images are produced in black-white format; each white dot in the image representing a reflected sound wave. Sound waves travel in a similar way to light waves and hence the denser a material is (e.g. bone cortex) the more reflective it becomes and accordingly, the whiter it appears on the screen. On the other hand, water is the least reflective tissue and therefore it appears as black while the sound waves travel straight through it. Two factors influence reflectivity: The acoustic impedance of materials and the angle of incidence of the sound beam. Acoustic impedance is the product of a material density and the speed of sound within that substance. According to the intensity of the echo, images are categorized in three forms. Anechoic: A structure that does not produce any internal echoes. Hypoechoic: A term used to describe an area that has decreased brightness of its echoes relative to an adjacent structure. Hyperechoic: A term used to describe a structure which has increased brightness of its echoes relative to an adjacent structure.

The transducer is an essential part of the US equipment and is responsible for the generation of the US beam and the detection of returning echoes. A variety of linear-array transducers, including large (> 40 mm), medium-sized (< 40 mm) and small-field of view (hockey-stick-shaped) probes, are currently available in the frequency range used for musculoskeletal examinations. Selection of the most appropriate transducer primarily depends on the frequency (multifrequency, high frequency, low frequency, etc.) whereby high-frequency probes (e.g. 10-18 MHz) are used to visualize superficial structures and low-frequency ones (e.g. 5-10 MHz) for deeper tissues.

The improvement in fast digital computer processing and memory storage capacity have recently improved the possibility of applying 3-Dimensional technology to US. 3D acquisition can be achieved with US using either 2D conventional transducers equipped with a small electromagnetic positional sensor or dedicated "3D-volume transducers" which are larger than standard probes. Although it is difficult to handle those probes, they provide better assessment of each scanning plane.

Newer US techniques, including color and power Doppler imaging, provide color maps of tissues. The amount of color is related to the degree of blood flow, which may be of use in the assessment of vascular tissues as in soft tissue inflammation [5].

In this review, we will focus on major topics whereby MSUS has improved our diagnostic, interventional and follow-up abilities in pediatric rheumatology.

### Juvenile idiopathic arthritis

Juvenile idiopathic arthritis (JIA) is the most common chronic inflammatory arthropathy in childhood, accounting for approximately 6-19 cases per 10^6 ^children per year [6]. It is a heterogeneous group of disorders, the majority of which are different from adult seropositive rheumatoid arthritis [7]. It is characterized by arthritis that persists for a minimum of 6 consecutive weeks in one or more joints, commencing before the age of 16 years. Herewith, the roster of differential diagnoses encompasses several conditions that display joint inflammation [8,9].

Similar to the stituation in adult rheumatoid arthritis, MSUS has proven to be valuable in the early diagnosis of JIA, for evaluation and follow-up of disease activity and for the assessment of treatment response [10]. It is exquisitely sensitive in detecting synovitis, intra-articular effusion, and cartilage edema/thinning or bony erosions [11,12].

#### Synovitis

The synovial membrane is an important connective tissue lining the inner surface of the joint capsule, tendon sheath, and bursa. Therefore, it is essential to understand the pathogenesis and the pathological changes seen in inflammatory synovium in order to perform a complete scan of the synovial joints. In JIA, as in any other inflammatory arthritis, the synovium undergoes significant changes leading to the formation of a mass of synovial tissue. This is the result of edema, multiple redundant folds, and villae. The presence of joint, bursal or tendon sheath effusion is used as an excellent, indirect correlate of synovial inflammation. Further, its presence (as an anechoic structure) technically enables a better visualization of the synovial thickening, proliferation and villous formation during MSUS imaging [13].

In the absence of an effusion, synovitis is diagnosed by the presence of an abnormally thickened hypoechoic region, usually measured in a standard plane with reference to an established normal range or to the contralateral normal joint. Therefore, MSUS can easily detect significant degrees of synovitis which is not determined by clinical examination [14,15] and can reliably discriminate inflammatory and noninflammatory joint disease. Moreover, the detection of subclinical synovitis may also lead to re-evaluation of the clinical classification of arthritis as oligoarticular or polyarticular.

With MSUS, synovial hypertrophy is detected as solid, non-compressible, hypoechoic tissue in connection to joint lines or surrounding tendons [16]. In children, detection is more challenging than in adults as the synovial tissue is often difficult to distinguish from the hypoechoic cartilage of epiphyses. To avoid diagnostic errors, it is therefore important to have good knowledge of the age-dependent normal MSUS appearance of each joint.

Evaluation has been enhanced on machines with power Doppler setting which depicts the increased vascularity of the hypertrophied synovium by demonstrating microvascular flow. The Doppler signal can distinguish between active and inactive synovitis, correlating to clinical and laboratory data [17-19], MRI (20) and histology as well [21]. Power Doppler also shows promise in evaluating the amount and the activity of pannus in JIA. Yet, proliferative synovium, which is extremely vascular, shows high power Doppler signal [7].

#### Intra-articular Fluid

Athough it is nonspecific, joint effusion is a valuable indicator of active joint disease. US has been shown to be one of the best methods for detection of increased intra-articular fluid and synovial proliferation. Graded compression is useful in distinguishing isolated effusion from synovial proliferation. Effusions, as small as 1 mL can be detected with ultrasound and interobserver agreement for ultrasound detection of effusion in hand and foott joints is reported to be 79% [22].

Using MSUS to detect and localize small joint effusions is effective in clinical practice. In patients with inflammed MCP and proximal interphalangeal (PIP) joints, MSUS improved accurate needle placement from 59% by palpation guidance to 96% by MSUS guidance [23]. MSUS has been confirmed to be superior to clinical examination in the detection of effusion, even in a large and relatively easily palpable joint such as the knee joint [24]. However, it cannot yet accurately differentiate whether a fluid collection is inflammatory, infectious or hematogenous in most cases and aspiration of fluid --which is more successful with MSUS guidance-- remains the gold standard. MSUS can give a basic estimate of fluid viscosity, aiding selection of the appropriate gauge size of the needle for fluid aspiration. Finally, it is important to appreciate that some types of effusions, such as high-pressure echogenic effusion, can be mistaken for synovitis as the fluid will appear hyperechoic and is not easily displaceable by the probe [13].

#### Cartilage Alterations

In children with JIA, MSUS imaging has been shown to be a sensitive modality to detect alterations in the articular cartilage [25]. It allows direct visualization of articular cartilage that is normally seen as an anechoic structure with a smooth outline over the bone surfaces. Cartilage edema or loss may be seen in rheumatoid diseases according to the level of the condition [25].

Cartilage edema in early stages of JIA can be detected sonographically as thickening of the articular cartilage. Chronic inflammation of the cartilage results in permanent damage to the articular surface. This is observed sonographically as blurring of the articular surface. Continued destruction of the cartilage due to rheumatoid disease is seen as pitting of the articular surface and measurable thinning of the cartilage. Cartilage loss is better detected than on plain films especially in young children with thick epiphyseal cartilage and at the early stages of the disease [6].

In a cohort of healthy children of different ages, the reliability of the assessment of cartilage thickness with US has been recently demonstrated by Spannow et al [26]. The authors found a good intra- and inter-observer agreement both in large and small joints, using ultrasonographic standard scans according to EULAR guidelines [26]. Furthermore, Moller et al. [27] have recently demonstrated that direct visualization and quantification of cartilage in MCP and PIP joints by using MSUS is objective, reliable and valid. They have suggested its use for diagnostic purposes in rheumatoid patients.

The presence of juxta-articular flow at color Doppler examination in the growing child may either represent normal flow of the well-vascularised cartilage of the epiphysis or synovial hyperemia indicating inflammation. Flow in the cartilage probably indicates normal cartilaginous flow in contrast to flow inside the synovium which probably indicates hyperemia [19].

#### Bone Erosions

Studies assessing long-term outcome of JIA have shown that a relevant proportion of patients may develop progressive joint destruction and serious physical disability [28]. The development of erosions early in the disease course has been associated with a higher risk of progressive disease and has been included among the poor prognostic indicators of long-term outcome [29,30]. Conventional radiography is quite insensitive as it usually reveals late and often irreversible signs of bone erosion. These signs can be important in making a diagnosis of inflammatory arthritis as well as monitoring disease activity and joint damage that can help guide therapy [31,32].

In line with previous literature concerning rheumatoid arthritis, recent studies on JIA confirmed that MSUS is equal or superior to plain radiography in detecting cortical erosions in sonographically accessible areas, but that it is less reliable in detecting intramedullary lesions and those within the centres of larger joints, due to the acoustic shadowing from overlying bones [6,28,33].

Bone is often regarded as a barrier to the use of MSUS in clinical practice. On the other hand, one of the most exciting applications of MSUS is the evaluation of bone erosions in rheumatologic diseases. On MSUS, bone erosions can be seen as interruption of the smooth, continuous hyperechogenic line corresponding to the bony cortex. Marginal erosions are usually seen in rheumatoid diseases and are identified as crater-like defects in the bony contours along the edges of the articular cartilage. Recent studies support the superiority of MSUS over radiography in detection of erosions [34,35]. Experienced ultrasonographers can identify them nearly as well as MRI, and notably with lower cost and more efficiency [36].

### Other pediatric rheumatic diseases

#### Juvenile Spondyloarthropathies

**Figure 1:**
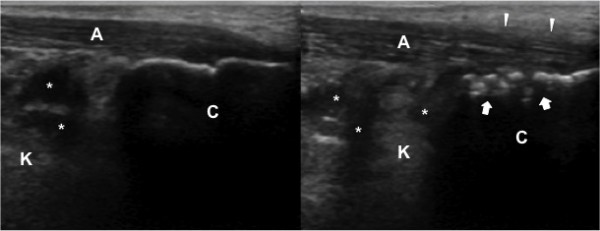
**A 13-year-old boy with bilateral heel pain who was eventually diagnosed as enthesitis-related arthritis**. Comparative ultrasound evaluations (longitudinal view) demonstrate increased thickness, and edema of the right achilles tendon (A). The paratenon is blurred *(white arrow heads) *and there are irregularities *(white arrows) *at the insertion site on calcaneus (C) (right image). The echogenicity of Kager's fat pad (K) is also irregular on both sides.

**Figure 2:**
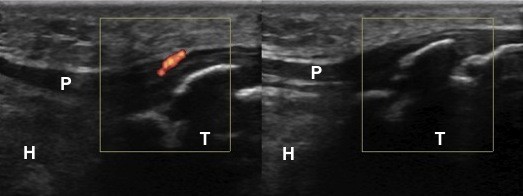
**A 10-year-old girl with unilateral knee pain who was eventually diagnosed as bilateral Osgood-Schlatter disease and patellar tendinitis on the symptomatic side**. Comparative power Doppler ultrasound evaluations (longitudinal view) demonstrate cortical irregularities at the insertion site of the patellar tendon (P) on the tibia (T) bilaterally. There is also abnormal power Doppler signal on the patellar tendon (left image). The Hoffa's fat pad (H) is normal on both sides.

Juvenile spondyloarthropathies which form the second most common form of chronic arthritis in children, are a group of disorders that affect the axial and extra-axial joints [32,37]. Juvenile ankylosing spondylitis, reactive arthritis, and arthritis associated with inflammatory bowel disease are all seen in children younger than 16 years of age, but joint findings are generally limited [38]. Synovitis and enthesitis (inflammation at the site of attachment of ligaments or tendons to bone) are major types of inflammation in this group of patients. Enthesitis is most commonly present at the insertions of the achilles tendon, plantar fascia, and the patellar and quadriceps tendons (Figures 1 &2). Soft tissue swelling, localized osteopenia, bone erosions or spurs are commonly observed.

MSUS is useful for detection of synovial effusions/hypertrophy and various forms of enthesopathy, i.e. calcifications, enthesophytes, bony erosions at insertion sites. It may also show loss of the normal fibrillar echogenity of tendons, absence of the homogeneous pattern, blurring of tendon margins, and irregular fusiform thickening [39]. The ability of power Doppler sonography to assess low-velocity blood flow in the synovium allows a clear depiction of minimal increases of perfusion in spondyloarthropathy [32,40].

#### Systemic Lupus Erythematosus

Musculoskeletal involvement of systemic lupus erythematosus includes arthralgia and arthritis/synovitis typically affecting the small joints of the hands, wrists and knees. It is generally not erosive, but can be deforming [41]. MSUS can detect fluid within the synovial sheath of the tendons, synovial thickening, and partial/complete tendon ruptures [42]. It can easily be used as the first imaging modality to evaluate children with clinical suspicion of tenosynovitis or bursitis in systemic lupus erythematosus [43].

### Future

There are some innovations regarding ultrasound technology like sono-elastography [44]. It is a non-invasive method in which stiffness or strain images of soft tissue are used to detect or classify mass lesions. Other recent advances also include new technologies that combined with MRI and high-intensity focused ultrasound for confirmative diagnosis [45].

## Conclusion

MSUS is suitable for examination of children of all ages and it has certain advantages over other imaging modalities [46,47]; it is cheaper, mobile, instantly accessible bedside, easy to combine with clinical assessment (interactivity) and non-invasive. It does not require sedation, which facilitates repetitive examinations. Assessment of multiple locations is possible during the same session. Agitation is rarely a problem and small children can be seated in their parents' lap or they can even play while being examined. In this regard, when compared with the (already established) role of MSUS in the daily practice of adult musculoskeletal medicine, it is time for pediatric rheumatologists to start to use MSUS as well.

## List of abbreviations

MSUS: Musculoskeletal Ultrasound; JIA: Juvenile Idiopathic Arthritis; MRI: Magnetic Resonance Imaging

## Competing interests

The authors declare that they have no competing interests.

## Authors' contributions

FT, ED and LO have made substantial contributions to conception and design.

FT, ED and LO have been involved in drafting the manuscript or revising it critically for important intellectual content.
